# Sepsis accelerates frailty and functional decline in older adults: a 12-month prospective study

**DOI:** 10.1007/s11739-026-04330-0

**Published:** 2026-03-30

**Authors:** Ian López-Cruz, José Luis García-Giménez, Laura Piles, Judit Garcia, Manuel Madrazo, Federico Vicente Pallardó, Arturo Artero

**Affiliations:** 1 Department of Internal Medicine, Dr. Peset University Hospital, Valencia, Spain; 2 Department of Medicine, Faculty of Medicine and Dentistry, University of Valencia, Valencia, Spain; 3 Department of Physiology, Faculty of Medicine and Dentistry, University of Valencia, Valencia, Spain; 4 INCLIVA Biomedical Research Institute, Valencia, Spain; 5 Center for Biomedical Network Research on Rare Diseases (CIBERER), Institute of Health Carlos III, Valencia, Spain; 6 EpiDisease S.L., Parc Científic de la Universitat de València, Paterna, Spain

**Keywords:** Sepsis, Frailty, Functional capacity, Activities of daily living, Comorbidity, Long-term outcomes

## Abstract

Sepsis, a life-threatening infection-induced condition, is a leading cause of mortality worldwide, particularly among older adults. Survivors often experience long-term health complications, including decline in functional capacity, cognitive impairment, and worsening of chronic comorbidities. This study aimed to evaluate the impact of sepsis on frailty and functional status over 12 months in patients with community-acquired infections. 50 patients completed the follow-up and were analyzed: 24 in the sepsis group and 26 in the control group, matched by age, sex, and infection source. Functional status was assessed at baseline and 12 months using the Barthel Index, Lawton Scale, and FRAIL questionnaire. At 12 months, septic patients showed significant declines in both functional capacity and frailty. Specifically, there was a significant decline in functional independence as measured by the Lawton Scale, with 33.3% of septic patients exhibiting reduced ability in instrumental activities of daily living, compared to 11.7% in controls (*p = *0.032). FRAIL scale showed significant worsening in the sepsis group (41.7% vs. 7.7%, *p = *0.005). Notably, 33.3% of previously robust septic patients developed new frailty, compared to 0% in the control group (*p = *0.025). The study also found a significant increase in comorbidity burden at 12 months within the sepsis group (*p = *0.041), whereas between-group differences were not statistically significant. These results highlight the long-term impact of sepsis on functional capacity and frailty, emphasizing the need for targeted post-sepsis rehabilitation and management.

**Supplementary Information:**

The online version contains supplementary material available at 10.1007/s11739-026-04330-0.

## Introduction

Sepsis is a life-threatening clinical condition initiated by infection and characterized by an abnormal and dysregulated host immune response. It can progress to multi-organ dysfunction and remains a major contributor to global mortality [1, 2]. Beyond its high acute-phase mortality, sepsis is frequently linked to adverse long-term outcomes, including elevated late mortality. Although the majority of deaths occur within the first 6 months, an increased risk persists for up to 2 years following the initial episode [3].

The incidence of sepsis rises with age. Over half of all sepsis cases occur in individuals over 65 years old, a group that also exhibits increased infection-related mortality [4]. Several factors, such as frailty, malnutrition, chronic comorbidities, and cognitive impairment, have been proposed as potential contributors to an increased risk of sepsis and poorer outcomes during the acute phase [5, 6].

Survivors of sepsis may also experience a decline in physical activity and functional capacity, along with quality of life and cognitive impairments, and the development of new or worsening chronic health conditions [7–9]. Following hospitalization for sepsis, patients face an elevated risk of ongoing health decline, readmission, and late mortality, outcomes that are not fully explained by age or preexisting comorbidities [10, 11]. Evidence on factors influencing long-term prognosis after sepsis remains limited. Some studies identify comorbidities and baseline functional status as major determinants of mortality and physical decline [8, 12].

Frailty is a multidimensional clinical syndrome defined by reduced physiological reserve and diminished resilience to stressors, representing a state of increased vulnerability to adverse health outcomes such as falls, disability, hospitalization, and mortality [13]. Frailty is considered a potentially preventable and reversible condition. While some studies suggest that preexisting frailty contributes to poorer long-term outcomes in sepsis [14], the impact of sepsis on the progression of frailty over time and the capability to predict frailty trajectories measured by standardized frailty tools remains insufficiently explored. A recent narrative review summarized how sepsis could precipitate frailty through cognitive, functional, and physiological decline, particularly in older adults, and emphasized the need for structured follow-up to improve recovery [15].

Although targeted post-sepsis therapies are lacking, integrated follow-up strategies have shown potential to improve long-term outcomes [16]. Understanding the trajectory of sepsis and frailty could aid in the early identification of high-risk patients and support the development of targeted management strategies to improve clinical outcomes.

Our objective was to evaluate the impact of sepsis on patients’ frailty and functional status using a one-year prospective cohort study. This involved comparing patients with infections who developed sepsis to a control group of patients with similar infections who did not progress to sepsis, matched for age and sex.

## Materials and methods

### Study design and patient selection

This prospective cohort study included patients aged ≥ 18 years who were admitted to the internal medicine ward of a university hospital between September 2019 and December 2022, and were followed for 1 year after hospital discharge until December 2023.

Patients were included in the study if they were admitted to hospital with community-acquired urinary tract infections (UTI), community-acquired pneumonia (CAP), or abdominal infections, and either presented with sepsis upon admission or had infections of the same focus without developing sepsis; the latter group served as the control group.

Sepsis was diagnosed according to the Third International Consensus Definitions for Sepsis and Septic Shock (Sepsis-3) [17]. The final classification as septic or non-septic was confirmed within the first 24 h of admission, once complete clinical and laboratory data were available. To enhance comparability between groups, patients were matched based on sex, age (± 5 years), and source of infection. To minimize selection bias, enrollment was conducted consecutively by order of admission, with each non-septic control manually matched to the first septic patient fulfilling these criteria. Exclusion criteria comprised severe functional dependency (Barthel Index < 40), immunocompromised status, poor short-term prognosis due to preexisting conditions, and infections of nosocomial origin.

Clinical and epidemiological data were obtained through patient interviews and review of electronic medical records. Preexisting comorbidities were evaluated using the age-adjusted Charlson Comorbidity Index (CCI) [18].

The nutritional risk of participants was assessed using a validated screening tool, the Mini Nutritional Assessment–Short Form (MNA-SF) [19].

Functional status and frailty were assessed at baseline and reassessed 12 months later using validated instruments. Functional status refers to an individual’s capacity to independently perform daily activities, encompassing both basic and instrumental activities of daily living [20]. Frailty is a clinical syndrome characterized by decreased physiological reserves and increased vulnerability to stressors [13]. For this study, frailty was assessed using the FRAIL (Fatigue, Resistance, Ambulation, Illnesses and Loss of weight) questionnaire, selected for its simplicity, validation in hospitalized older adults, and feasibility for repeated assessments during acute illness and post-discharge follow-up [21, 22]. Functional status was measured by the Barthel Index for basic activities of daily living (ADL), and the Lawton and Brody Scale for instrumental activities of daily living (IADL) [23, 24].

All patients received standard clinical care throughout the study. They were followed for 12 months through in-person visits, during which they underwent clinical reassessment, and the same scales were repeated to monitor any changes in their condition.

The study was conducted in accordance with the principles outlined in the Helsinki Declaration (Fortaleza version, 2013), as well as with Spanish regulations on Personal Data Protection (15/1999) and Biomedical Research (14/2007). It was approved by the Ethics Committee of “Doctor Peset University Hospital” (Code: 73/19). Written informed consent was obtained from all participants.

### Statistical analysis

Statistical analysis was conducted using IBM SPSS Statistics version 22. Normality of the data was assessed with the Shapiro–Wilk test. For normally distributed variables, means and standard deviations were calculated, whereas medians and interquartile ranges (IQR) were used for non-normally distributed variables. Qualitative variables were expressed as frequencies and percentages. Comparisons between groups were performed using Student’s *t* test for parametric data, and the Mann–Whitney *U* test for non-parametric data. For categorical variables, Chi-square and Fisher’s exact tests were applied. The Wilcoxon signed-rank test was employed in this study to assess the differences in the frailty and dependency scales (FRAIL scale, Barthel Index and Lawton Scale) at 12 months compared to baseline scores. This non-parametric test was chosen because the data consisted of paired observations, and the assumption of normality for the paired differences could not be confirmed.

To evaluate potential attrition bias, we compared baseline characteristics between patients who completed the 12-month follow-up (completers) and those who did not (non-completers). Continuous variables were compared using the Mann–Whitney *U* test, and categorical variables using *χ*^2^ or Fisher’s exact test, as appropriate.

## Results

**Fig. 1:**
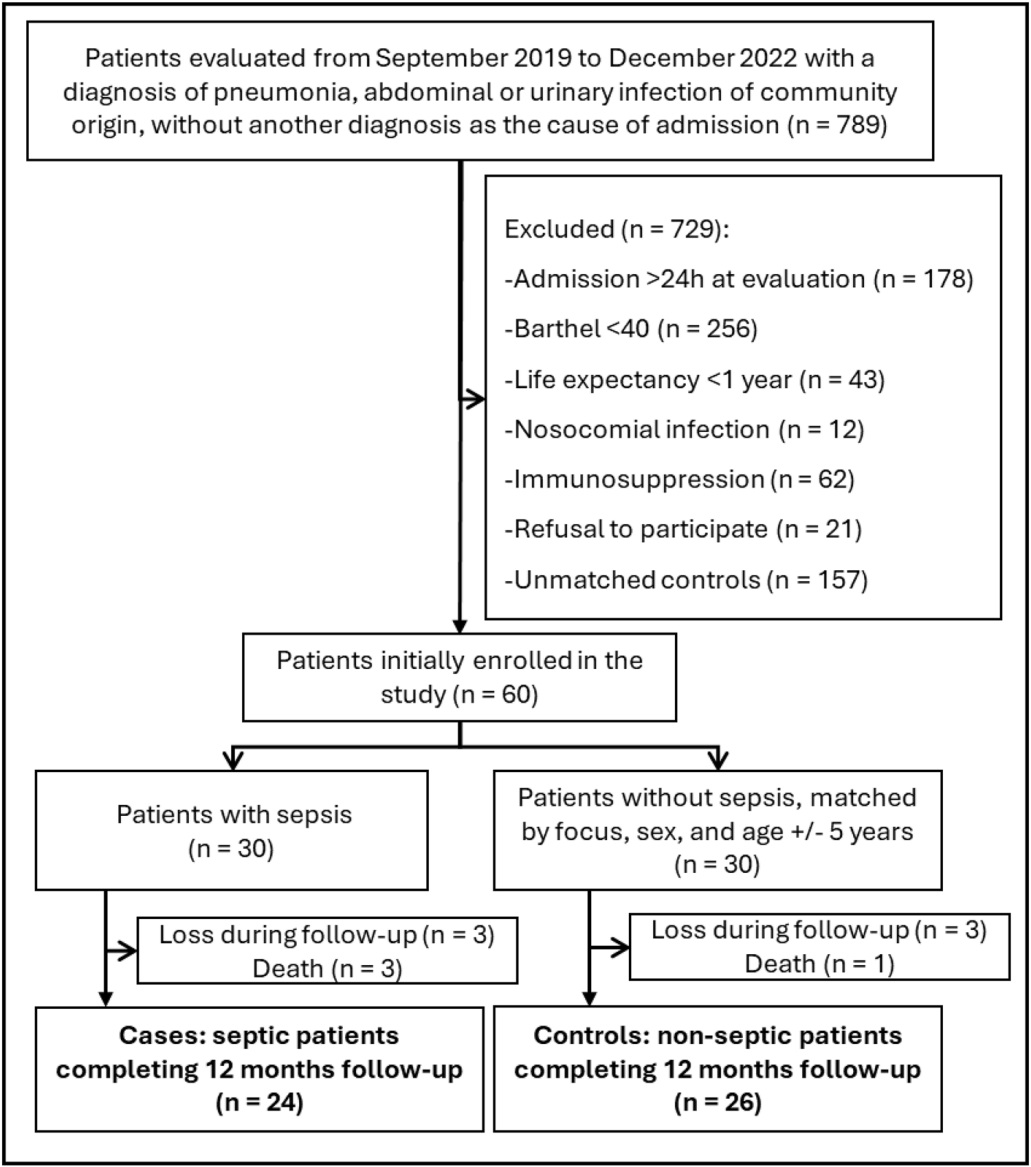
Flowchart of patient enrollment and follow-up

A total of 789 patients were screened during the study period. Of these, 60 patients fulfilled the eligibility criteria and were included in the study. Six patients were lost to follow-up after hospital discharge and four patients died. Therefore, 50 patients were ultimately analyzed. The patient inclusion and exclusion criteria are detailed in Fig. 1.

Women accounted for 56.6% of the sample, and most individuals (88.3%) were aged between 61 and 90 years (median age: 78 years, IQR: 68–84). The most frequent source of infection was the urinary tract (76.7%), followed by community-acquired pneumonia (20.0%) and cholangitis (3.3%).

CCI scores showed a high proportion of patients with severe comorbidity in both groups, with a median of 5 points, and a high prevalence of polypharmacy at admission. The most common comorbid conditions were diabetes mellitus (31.7%), chronic obstructive pulmonary disease (21.7%), chronic kidney disease (16.7%), and heart failure (15%). Comorbidity profiles were comparable between groups at the time of admission, except for a history of prior malignancy, which was more frequently reported in the sepsis group (20% vs. 1%, *p = *0.035). Nutritional risk screening using MNA-SF revealed significant group differences, with patients in the sepsis group demonstrating a substantially higher risk of malnutrition compared to controls (43.3% vs. 16.7%; *p = *0.024). As expected, there were significant differences between groups in qSOFA, SOFA, and APACHE II scores, as well as in the occurrence of septic shock, though this difference was not statistically significant. Baseline characteristics, nutritional assessment and severity scales are presented in Table 1.

**Table 1:** Characteristics, comorbidities and severity scales at admission

	Septic patients (*n = *30)	Non-septic patients (*n = *30)	*p* value
Age (years), median [IQR]	78 [66–87]	76,5 [68–82]	0.796
Female sex, *n* (%)	17 (56.7)	17 (56.7)	1.000
Comorbidities, *n* (%)			
Stroke	1 (3.3)	1 (3.3)	1.000
Asthma	1 (3.3)	5 (16.7)	0.073
Dementia	1 (3.3)	1 (3.3)	0.236
Diabetes mellitus	9 (30)	10 (33.3)	0.781
Coronary artery disease	3 (10)	4 (13.3)	0.687
Chronic kidney disease	4 (13.3)	6 (20)	0.488
Connective tissue disease	2 (6.7)	1 (3.3)	0.550
Chronic obstructive pulmonary disease	4 (13.3)	9 (30)	0.113
Chronic liver disease	1 (3.3)	2 (6.7)	0.550
Heart failure	6 (20)	3 (10)	0.274
Neoplasm	6 (20)	1 (3.3)	**0.035**
Peptic ulcer	1 (3.3)	3 (10)	0.290
Peripheral vascular disease	2 (6.7)	1 (3.3)	0.550
Charlson Comorbidity Index, median [IQR]	5 [4–6.25]	4.5 [4–6.25]	0.782
Polypharmacy ≥ 5 drugs, *n* (%)	20 (66.7)	19 (63.3)	0.787
MNA Scale, *n* (%)			
Normal nutritional status: 12–14 points	17 (56.7)	24 (80)	0.052
Malnutrition risk: 8–11 points	13 (43.3)	5 (16.7)	**0.024**
Malnutrition: ≤ 7 points	0	1 (3.3)	0.236
Severity scales			
APACHE-II, mean ± SD	13.6 (± 2.9)	7.4 (± 3.2)	** < 0.001**
qSOFA ≥ 2, *n* (%)	26 (86.7)	0	** < 0.001**
SOFA, median [IQR]	4 [3–5]	0 [0–1]	** < 0.001**
Septic shock, *n* (%)	3 (10)	0	0.236

*MNA* mini nutritional assessment

No significant differences were found between the groups in terms of pre-admission frailty or functional dependence. Barthel Index revealed that 42 patients (70%) were independent, 16 (26.7%) had mild dependence, and 2 (3.3%) showed moderate dependence. Based on the FRAIL scale, 23 individuals (38.3%) were categorized as robust, 19 (31.7%) as pre-frail, and 18 (30%) as frail at admission. Functional status and frailty at admission and 1 year after hospital discharge are detailed in Table 2.

**Table 2:** Functional and frailty status at admission and one year after hospital discharge

	Admission			1-year follow-up		
	Septic (*n = *30)	Non-septic patients (*n = *30)	*p* value	Septic patients (*n = *24)	Non-septic patients (*n = *26)	*p* value
Barthel Index, *n* (%)						
Independence ADL: 100 points	21 (70)	21 (70)	1.000	17 (70.8)	18 (69.2)	0.902
Mild dependence ADL: 60–95 points	8 (26.7)	8 (26.7)	1.000	5 (20.8)	6 (23.1)	0.848
Moderate dependence ADL: 40–55 points	1 (3.3)	1 (3.3)	1.000	0 (0)	2 (7.7)	0.166
Severe dependence ADL 12 m: < 40 points	–	–	–	2 (7.7)	0 (0)	0.133
Lawton Scale, *n* (%)						
Independence IADL: 8 points	14 (46.7)	16 (53.3)	0.606	11 (45.8)	13 (50)	0.768
Mild–moderate dependence IADL: 4–7 points	14 (46.7)	11 (36.7)	0.432	8 (33.3)	8 (30.8)	0.846
Severe dependence IADL: 0–3 points	2 (6.7)	3 (10)	0.640	5 (20.8)	5 (19.2)	0.997
FRAIL Scale, *n* (%)						
Robust health status: 0 points	9 (30)	14 (46.7)	0.184	7 (29.2)	13 (48.1)	0.166
Pre-frail: 1–2 points	12 (40)	7 (23.3)	0.165	5 (20.8)	7 (25.9)	0.669
Frail: 3–5 points	9 (30)	9 (30)	1.000	12 (50)	7 (25.9)	0.076

*ADL* activities of daily living; *FRAIL* fatigue, resistance, aerobic capacity, illnesses, and loss of weight; *IADL* instrumental activities of daily living

The potential influence of attrition bias was examined by comparing baseline characteristics between patients who completed the 12-month follow-up and those who did not. As shown in Supplementary Table S1, completers (*n = *50) and non-completers (*n = *6) had broadly similar demographic and clinical profiles, with only slightly higher rates of chronic kidney disease and peripheral vascular disease in the latter. The prevalence of sepsis was similar between groups (48% vs 50%, *p = *0.93).

Septic patients had a significantly longer hospital stay compared to controls, with a median of 5.5 days [IQR 4–9.25] versus 3 days [IQR 2–5.25] (*p = *0.001). Eight patients required ICU admission, with 7 (23.3%) in the sepsis group and 1 (3.3%) in the non-sepsis group (*p = *0.052). The median ICU stay was 3 days [IQR 2–4.75]. A total of 4 patients died during the study, 3 of them in the sepsis group (10%) and 1 in the control group (3.3%). One patient from each group died during hospitalization, while the others died during the follow-up within the first 3 months. The death of the non-septic patient was attributed to nosocomial pneumonia, which developed during the initial hospitalization for a non-septic urinary tract infection, and was complicated by sepsis and organ failure, requiring ICU admission.

**Fig. 2:**
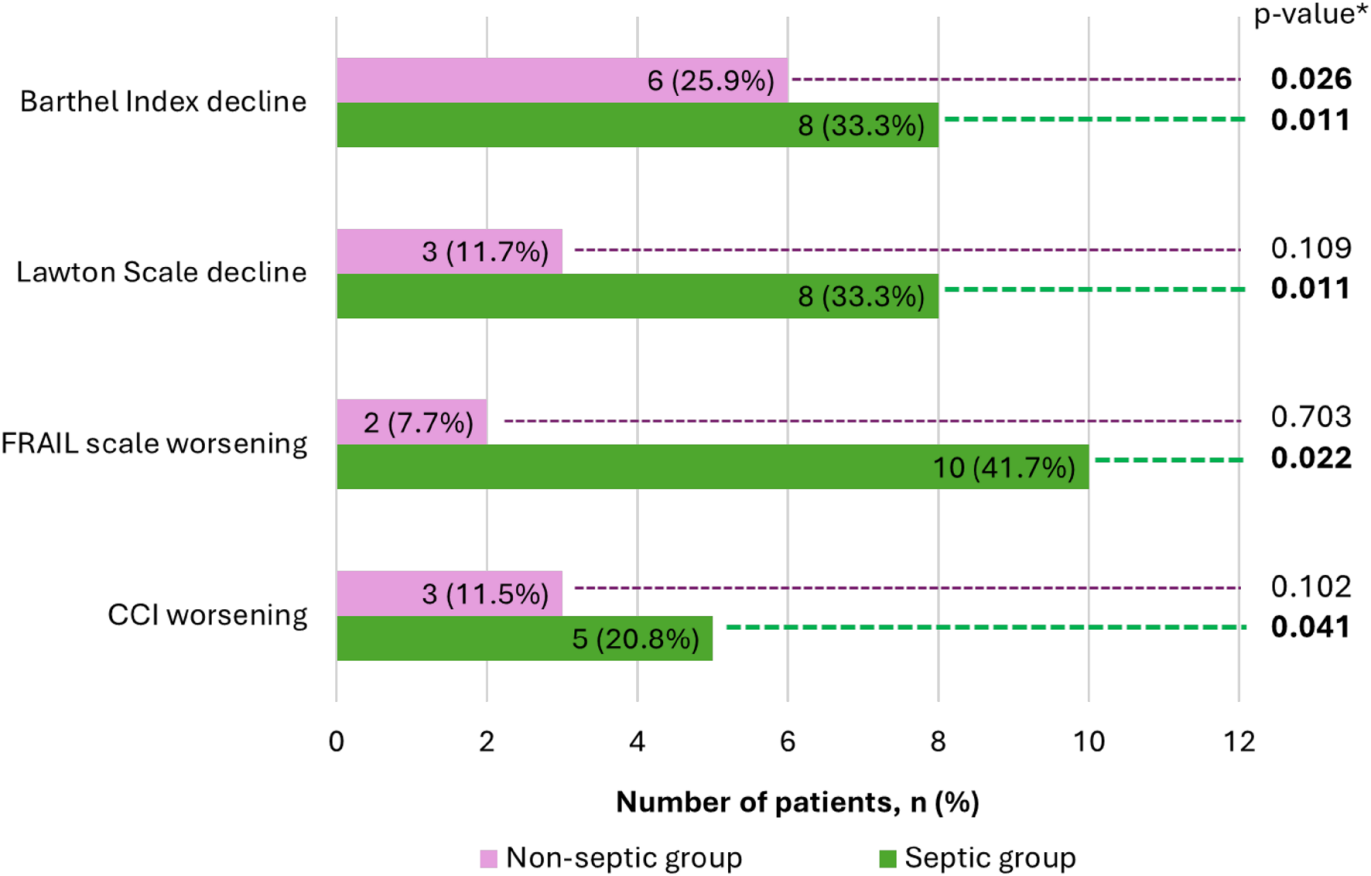
Number of patients with a change in functional status, frailty, and comorbidities scores one year after hospital discharge. *CCI* Charlson Comorbidity Index; *FRAIL* fatigue, resistance, aerobic capacity, illnesses, and loss of weight. *Wilcoxon signed-rank test *p* value (baseline vs. 12 months

Among the 50 patients who completed the follow-up, a statistically significant decline in functional status and frailty was observed at 1 year in the overall cohort, while comorbidity burden increased significantly only within the sepsis group. When changes in the scales were analyzed by subgroup, the sepsis group showed deterioration across all scales, whereas the non-sepsis group exhibited decline only in the Barthel Index. Specifically, the Barthel and Lawton scales showed deterioration in 8 patients in the sepsis group (*p = *0.011) with no cases of improvement. The FRAIL scale worsened in 10 septic patients (*p = *0.022) and 5 patients showed comorbidities worsening according to the CCI (*p = *0.041), indicating a significant increase in comorbidity burden within the sepsis group. In contrast, patients in the non-sepsis group only showed a decline in Barthel index at 1 year. The results are presented in Fig. 2.

When both groups were compared, septic patients demonstrated a more frequent deterioration in functional status, frailty scores, and comorbidities. Activities of daily living, measured by the Lawton scale, showed a greater decline among septic patients than in non-septic patients (33.3% vs 11.7%, *p = *0.032); although no significant differences were found between groups in Barthel Index scores. Similarly, patients with sepsis exhibited a higher prevalence of frailty, as evidenced by worsening FRAIL scores (41.7% vs 7.7%, *p = *0.005). New onset of frailty was observed in 6 previously robust septic patients, while no cases were reported in the non-sepsis group (33% vs 0%; *p = *0.019). An increased comorbidity burden (Charlson Comorbidity Index, CCI) was observed in both groups; but these differences did not reach statistical significance between them. These findings, summarized in Table 3, underscore the long-term burden of sepsis on patient independence and vulnerability.

**Table 3:** Changes in comorbidities, functional status, and frailty at one-year follow-up in septic and non-septic patients

	Overall cohort (*n = *50)	Septic patients (*n = *24)	Non-septic patients (*n = *26)	*p* value
Barthel decline at 12 months, *n* (%)	14 (28%)	8 (33.3)	6 (25.9)	0.562
Lawton Scale decline at 12 months, *n* (%)	11 (22%)	8 (33.3)	3 (11.7)	**0.032**
FRAIL Scale worsening at 12 months, *n* (%)	12 (24%)	10 (41.7)	2 (7.7)	**0.005**
New onset of frailty, *n* (%)^a^	6 (12%)	6 (33.3)	0 (0)	**0.025**
CCI worsening at 12 months, *n* (%)	8 (16%)	5 (20.8)	3 (11.5)	0.370

*CCI* Charlson Comorbidity Index; *FRAIL* fatigue, resistance, aerobic capacity, illnesses and loss of weight

^a^Percentage based on the total number of previously robust or pre-frail patients

Bold indicates statistical significance (*p* 0.05)

## Discussion

Our findings reveal that community-acquired sepsis is linked to reduced functional capacity and increased frailty 1 year after hospital discharge.

The design of our study aims to isolate the effect of sepsis itself on long-term outcomes. By comparing patients with sepsis to a control group rigorously matched for sex, age, and source of infection, and by excluding individuals with severe preexisting conditions associated with poor prognosis, we minimized potential confounding factors. Unlike previous studies that primarily described the incidence of frailty after ICU survival without a matched comparison group [25, 26], the novelty of our approach allows for a more precise attribution of the observed decline in functional status and onset of frailty to the sepsis episode 1 year after hospital discharge.

A notable feature of our cohort of patients with community-acquired sepsis was their advanced age, with a median age of 78 years and 85% being 65 or older, reflecting the greater susceptibility of older adults to sepsis [4]. Women accounted for 56.6% of the cohort, likely due to community-acquired urinary tract infections being the most common infection source [27]. The baseline characteristics and comorbidities were mostly similar between the sepsis and non-sepsis groups, except for a significantly higher prevalence of prior neoplasia (20% vs. 3.3%) and malnutrition risk (43.3% vs. 16.7%) in the sepsis group. Frailty and mild to moderate dependency for ADL were prevalent in both groups (30%), as well as dependency for IADL (near to 50%). These findings are consistent with previous studies [28–30], highlighting an increased vulnerability of this population to infection and sepsis development.

The association between frailty and adverse outcomes in septic patients is well-documented in the literature. Studies have consistently shown that frailty is linked to higher short-term and long-term mortality, increased length of hospital and ICU stays, and a significant reduction in quality of life [31]. For instance, a study using the Clinical Frailty Scale reported higher in-hospital mortality in frail patients compared to non-frail individuals, with an adjusted odds ratio of 2.00 (95% CI 1.39–2.89; *p < *0.001). Additionally, frail patients were less likely to be discharged home, underscoring the substantial impact of frailty on outcomes in critically ill patients [32]. Similarly, another study found that frailty was associated with poorer outcomes and increased resource use among older ICU patients with suspected infection [33]. While prior research has explored preexisting frailty as a prognostic factor in sepsis, few studies have assessed frailty onset post-sepsis and hospital discharge.

No significant differences between the groups in terms of functional status or frailty at baseline suggest that pre-hospital health conditions were comparable. However, 1 year after discharge, patients with sepsis experienced significantly greater deterioration in both functional independence and frailty. The statistically significant worsening observed in the Barthel Index, Lawton scale, and FRAIL scale across the entire cohort reflects a broader trend of post-hospital functional decline, occurring both in hospitalized patients with sepsis and in those with infection without sepsis. However, the more pronounced deterioration in sepsis survivors, particularly in IADLs and frailty, underscores the long-term impact of critical illness on older adults.

Notably, the decline was more pronounced in the Lawton scale than in the Barthel Index, indicating that sepsis predominantly affects instrumental rather than basic activities of daily living (IADLs vs. ADLs). This pattern suggests an early loss of functional complexity and autonomy in everyday tasks. These results are consistent with the findings of Iwashyna et al. [9], who reported significantly more new functional limitations in septic patients compared to non-septic controls (1.50 vs. 0.49 new limitations, *p < *0.001). Detecting small functional changes in ADL, however, may require larger sample sizes and longer follow-up periods. In contrast, more pronounced outcomes were evident in our cohort: new-onset frailty was observed exclusively in the sepsis group (33.3% vs 0%, *p = *0.019), highlighting the potential role of sepsis in accelerating biological aging and reducing resilience in previously robust individuals.

The pathophysiology of physical deterioration following sepsis is multifactorial and may involve a complex interplay of persistent inflammation, immunosuppression, and catabolism [34]. Sepsis survivors often experience a persistent myopathy marked by skeletal muscle atrophy, weakness, and impaired muscle regeneration. This myopathy is triggered by the interaction between circulating pathogens and impaired muscle metabolic status, exacerbated by prolonged physical inactivity during hospitalization [35]. These pathophysiological changes could explain the significant long-term physical impairment for sepsis survivors [10].

In this study, a longer hospital stay was observed among septic patients. They also showed a higher proportion of ICU admissions, although this difference did not reach statistical significance (*p = *0.052). The observed overall mortality rate of 6.6% was notably lower than previously reported figures in the literature, which range from 10 to 52% [6, 36]. This discrepancy may be explained by the study's inclusion criteria, which intentionally excluded individuals with preexisting conditions associated with limited life expectancy, thereby reducing potential bias and influencing overall survival outcomes. However, these strict inclusion criteria may also limit the generalizability of our mortality findings, as septic patients in real-world settings often present with greater baseline frailty and comorbidity. The small number of patients, particularly those requiring ICU admission, may limit the statistical power to draw definitive conclusions about these outcomes in our cohort.

There was also a statistically significant increase in comorbidity burden at one within the sepsis group (*p = *0.041), but not among non-septic patients. Consistently, some authors have noted a deterioration of preexisting chronic diseases or the development of new chronic conditions after a sepsis episode [3]. However, when comparing the proportion of patients with increased CCI between groups using the chi-square test (20.8% in septic vs. 11.5% in non-septic patients), the difference was not statistically significant. This suggests that, although within-group changes were significant among septic patients, the between-group difference in proportions was not large enough to reach statistical significance.

This study has some limitations. The most notable is the small sample size, influenced by strict inclusion criteria and challenges in recruitment during the COVID-19 pandemic. In addition, the single-center design and the high prevalence of UTIs may limit the generalizability of the results. Nevertheless, the rigorous patient selection process resulted in a highly homogeneous cohort, which likely reduced the impact of potential confounding variables and enhanced the reliability of the findings. Although cases and controls were matched on age, sex, and infection source, residual imbalances cannot be ruled out, and residual confounding may persist. The small number of patients lost to follow-up showed comparable baseline characteristics to completers, and the similar prevalence of sepsis across groups reduces the likelihood of attrition bias affecting long-term outcomes. No formal sample size calculation was performed due to the exploratory design, and the limited sample size may have reduced the power to detect small differences. Finally, the use of the FRAIL scale, while validated and feasible in acutely ill patients, may be less sensitive to subtle longitudinal changes and could limit comparability with studies using other frailty instruments. Overall, the results appear robust and provide valuable insights with potential significant implications for clinical practice.

It is noteworthy that current evidence shows that immune dysfunction and frailty reinforce one another, but an impaired immune system appears to be the primary driver. In this regard, immunosenescence, physiological stress, and “inflamm-aging” [37–39] lead to chronically elevated levels of pro-inflammatory cytokines and cortisol, which promote sarcopenia and erode physiological reserve, which are key steps in the emergence of the frailty phenotype. Importantly, sepsis can directly impact the epigenetic profile of immune cells, as we have found in previous studies [40, 41]. Therefore, although the impact of sepsis on the progression of frailty over time remains largely unexplored, exploring the epigenetic profile of immune cell populations may help to predict frailty trajectories [42, 43], also in sepsis survivors. In future studies, we aim to explore these epigenetic mechanisms to better understand their potential contribution to post-sepsis frailty.

Our findings highlight the long-term impact of sepsis on functional status and comorbidities, underscoring the need for targeted post-sepsis rehabilitation strategies. Future research with larger sample sizes and longer follow-up periods is essential to further understand the full impact of sepsis on long-term health outcomes.

## Conclusions

Sepsis appears to have a substantial impact on older adults, possibly accelerating the decline in functional status and contributing to the onset of new frailty, thereby underscoring the need for targeted follow-up in this vulnerable population.

## Supplementary Information

Below is the link to the electronic supplementary material.

Supplementary file1 (DOCX 16 KB)

## Data Availability

The datasets generated and/or analyzed during the current study are not publicly available due to privacy and ethical restrictions but are available from the corresponding author on reasonable request.
