# Supplementary material for: Sepsis accelerates frailty and functional decline in older adults: a 12-month prospective study
Source: Intern Emerg Med. 2026 Mar 30;21(5):1557–66. doi: 10.1007/s11739-026-04330-0 (PMC13452868; doi:10.1007/s11739-026-04330-0)
Supplement: Supplementary file 1 [file 11739_2026_4330_MOESM1_ESM.docx]

**Supplementary Table S1 Analysis of potential attrition bias: baseline characteristics of completers versus non-completers**

|  | **Completers**  **(n=50)** | **Non-completers (n=6)** | **p-value** |
| --- | --- | --- | --- |
| **Age (years), median [IQR]** | 76 [68-82] | 82 [80-86] | 0.248 |
| **Female sex, n (%)** | 30 (60) | 2 (33.3) | 0.385 |
| **Comorbidities, n (%)** |  |  |  |
| Stroke | 2 (4) | 0 (0) | 1 |
| Asthma | 4 (8) | 2 (33.3) | 0.119 |
| Dementia | 2 (4) | 0 (0) | 1 |
| Diabetes mellitus | 12 (24) | 0 (0) | 0.322 |
| Coronary artery disease | 6 (12) | 1 (16.7) | 0.569 |
| Chronic kidney disease | 6 (12) | 4 (66.7) | **0.007** |
| Connective tissue disease | 3 (6) | 0 (0) | 1 |
| Chronic obstructive pulmonary disease | 12 (24) | 1 (16.7) | 1 |
| Chronic liver disease | 3 (6) | 0 (0) | 1 |
| Heart failure | 5 (10) | 2 (33.3) | 0.158 |
| Neoplasm | 4 (8.0) | 0 (0) | 1 |
| Peptic ulcer | 3 (6.0) | 1 (16.7) | 0.372 |
| Peripheral vascular disease | 1 (2.0) | 2 (33.3) | **0.027** |
| **Charlson Comorbidity Index, median [IQR]** | 5 [4-6] | 6 [5-8] | 0.243 |
| **Polypharmacy ≥5 drugs, n (%)** | 30 (60) | 6 (100) | 0.078 |
| **MNA Scale, n (%)** |  |  |  |
| Normal nutritional status: 12-14 points | 36 (72) | 5 (83.3) | 1 |
| Malnutrition risk: 8-11 points | 14 (28) | 0 (0) | 0.319 |
| Malnutrition: ≤ 7 points | 0 (0) | 1 (16.7) | 0.107 |
| **Severity scales** |  |  |  |
| APACHE-II, mean ±SD | 10.5 (±4.5) | 10.3 (±4.6) | 0.852 |
| qSOFA ≥2, n (%) | 18 (36) | 3 (50) | 0.661 |
| SOFA, median [IQR] | 3 [1-4] | 3 [1-4] | 0.914 |
| Sepsis, n (%) | 24 (48) | 3 (50) | 0.93 |
| Septic shock, n (%) | 2 (4) | 0 (0) | 1 |

MNA: Mini Nutritional Assessment
